# European randomized study of prostate cancer screening: first-year results of the Finnish trial

**DOI:** 10.1038/sj.bjc.6690194

**Published:** 1999-03

**Authors:** L Määttänen, A Auvinen, U-H Stenman, S Rannikko, T Tammela, J Aro, H Juusela, M Hakama

**Affiliations:** Finnish Cancer Registry, Liisankatu 21 B, Helsinki, FIN-00170, Finland; Radiation and Nuclear Authority, Helsinki, Finland; Department of Clinical Chemistry, Helsinki University Central Hospital, Helsinki, Finland; Department of Urology, Helsinki University Central Hospital, Helsinki, Finland; Department of Urology, Tampere University Hospital, Tampere, Finland; Department of Surgery, Helsinki City Hospital, Helsinki, Finland; Department of Surgery, Jorvi Hospital, Espoo, Finland; Tampere School of Public Health, University of Tampere, Tampere, Finland

**Keywords:** screening, prostate neoplasms, randomized controlled trials, prostate-specific antigen

## Abstract

Approximately 20 000 men 55–67 years of age from two areas in Finland were identified from the Population Registry and randomized either to the screening arm (1/3) or the control arm (2/3) of a prostate cancer screening trial. In the first round, the participation rate in the screening arm was 69%. Of the 5053 screened participants, 428 (8.5%) had a serum prostate-specific antigen (PSA) concentration of 4.0 ng/ml or higher, and diagnostic examinations were performed on 399 of them. A total of 106 cancers were detected among them corresponding to a positive predictive value of 27%, which is comparable with mammography screening for breast cancer. The prostate cancer detection rate based on a serum PSA concentration of 4.0 ng ml^−1^ or higher was 2.1%. Approximately nine out of ten screen-detected prostate cancers were localized (85% clinical stage T1–T2) and well or moderately differentiated (42% World Health Organization (WHO) grade I and 50% grade II), which suggests a higher proportion of curable cancers compared with cases detected by other means. © 1999 Cancer Research Campaign


*British Journal of Cancer* (1999) **79**(7/8), 1210–1214
© 1999 Cancer Research Campaign
Article no. bjoc.1998.0194

### European randomized study of prostate cancer screening: first-year results of the Finnish trial

**L Määttänen[1], A Auvinen[1,2], U-H Stenman[3], S Rannikko[4], T Tammela[5], J Aro[6], H Juusela[6] and M Hakama[1,8]**

[1]Finnish Cancer Registry, Liisankatu 21 B, FIN-00170 Helsinki, [2]Radiation and Nuclear Authority, Helsinki, Finland; [3]Department of Clinical Chemistry, Helsinki University Central Hospital, Helsinki, Finland; [4]Department of Urology, Helsinki University Central Hospital, Helsinki, Finland; [5]Department of Urology, Tampere University Hospital, Tampere, Finland; [6]Department of Surgery, Helsinki City Hospital, Helsinki, Finland; [7]Department of Surgery, Jorvi Hospital, Espoo, Finland; [8]Tampere School of Public Health, University of Tampere, Tampere, Finland

**Summary** Approximately 20 000 men 55–67 years of age from two areas in Finland were identified from the Population Registry and randomized either to the screening arm (1/3) or the control arm (2/3) of a prostate cancer screening trial. In the first round, the participation rate in the screening arm was 69%. Of the 5053 screened participants, 428 (8.5%) had a serum prostate-specific antigen (PSA) concentration of 4.0 ng/ml or higher, and diagnostic examinations were performed on 399 of them. A total of 106 cancers were detected among them corresponding to a positive predictive value of 27%, which is comparable with mammography screening for breast cancer. The prostate cancer detection rate based on a serum PSA concentration of 4.0 ng $ml^{-1}$ or higher was 2.1%. Approximately nine out of ten screen-detected prostate cancers were localized (85% clinical stage T1–T2) and well or moderately differentiated (42% World Health Organization (WHO) grade I and 50% grade II), which suggests a higher proportion of curable cancers compared with cases detected by other means.

**Keywords:** screening; prostate neoplasms; randomized controlled trials; prostate-specific antigen

Prostate cancer is currently the most common cancer among men after skin cancer in most industrialized countries (Parkin et al, 1997). Because of its poorly understood aetiology, primary prevention is not currently feasible and therefore there is considerable interest in screening as a potential approach to prostate cancer control (Schröder and Boyle, 1993). The only means to establish the effect of screening is to conduct large randomized controlled trials with both mortality and quality of life as end points (Denis et al, 1995). Such studies are ongoing both in Europe and North America (Auvinen et al, 1996). In the absence of empirical results, a debate of the issue continues, which is demonstrated by the fact that so far the number of published reviews on the subject exceeds the number of original reports of randomized trials (as identified from Medline).

We report here the first-year results of the Finnish trial, which is part of the European Randomized Study of Screening for Prostate Cancer (ERSPC), a multi-centre randomized trial with participating centres from The Netherlands, Finland, Sweden, Belgium, Italy, Portugal and Spain. The common core protocol includes enrolment at ages 55–69 and use of Hybritech Tandem-E PSA assay as the screening test with a cut-off level of 4.0 ng $ml^{-1}$. The primary end point is prostate cancer mortality, with quality of life and cost-effectiveness as the secondary end points.

### MATERIALS AND METHODS

In the Finnish trial, the subjects are identified from the central population registry of Finland. Enrolment over 4 years with a total study population of 80 000 men is planned. The screening interval is 4 years and up to three screening rounds are planned. The primary end point is mortality from prostate cancer, which will be reported based on 10 years of follow-up from randomization.

The study population of the trial consists of men born in 1929–1944 and resident in the metropolitan areas of Helsinki and Tampere. In the first year of the study (1996), 20 398 men born in 1929, 1933, 1937 or 1941 were randomized. The subjects were identified from the Population Registry of Finland. The only exclusion criterion was a previous diagnosis of prostate cancer. Information on prostate cancer was obtained through record linkage with the Finnish Cancer Registry. A total of 90 men were excluded before randomization because of previously diagnosed prostate cancer. Approximately 1% of the population (200 men of the eligible study population) has forbidden the use of their address information for any purposes and they were also excluded from randomization. Each year, 8000 men, i.e. approximately one third of the eligible population, are randomized to the screening arm and the rest to the control arm. Of the 8000 men, men who had died or moved out of the study area between the date of randomization (in early January) and date of invitation, were not invited to participate (*n* = 388 in 1996). Due to an error, additional 275 men were not invited in 1996. Men in the control group were not contacted, but will be used as a reference group for assessment of cancer incidence and mortality at later stages. No information pertaining to the men in the control group was available for the present study. A letter of invitation explaining the purpose and procedures of the study was sent to the men in the screening arm. The letter also included information about occurrence of prostate cancer as well as risk factors and treatment options including their side-effects. In addition, the men were asked to fill in a questionnaire regarding urological symptoms and their treatment, as well as previous prostate-specific antigen (PSA) tests and family history of prostate cancer. After obtaining written informed

*Received 6 March 1998*
*Revised 9 September 1998*
*Accepted 25 September 1998*

*Correspondence to:* L Määttänen

**Table 1** Participation rate by age and centre in the Finnish prostate cancer screening trial

| | Invited | Participated | Participation rate (%) | |
|---|---|---|---|---|
| Age (years) | | | | |
| 55 | 2663 | 1798 | 68 | |
| 59 | 1812 | 1258 | 69 | |
| 63 | 1490 | 1045 | 70 | |
| 67 | 1372 | 952 | 69 | $P = 0.28$[a] |
| Centre | | | | |
| Helsinki | 5509 | 3707 | 67 | |
| Tampere | 1828 | 1346 | 73 | $P < 0.001$[a] |
| Total | 7337 | 5053 | 69 | |

[a]Statistical significance from a Pearson $\chi^2$ test.

**Table 2** Distribution of subjects by PSA concentration and age in the Finnish prostate cancer screening trial

| | Serum PSA concentration (ng ml$^{-1}$) | | | | | |
|---|---|---|---|---|---|---|
| | 0–1.9 *N* (%) | 2.0–2.9 *N* (%) | 3.0–3.9 *N* (%) | 4.0–9.9 *N* (%) | ≥10 *N* (%) | Total *N* |
| Age (years) | | | | | | |
| 55 | 1484 (83) | 176 (10) | 65 (4) | 61 (3) | 12 (1) | 1798 |
| 59 | 972 (77) | 138 (11) | 60 (5) | 70 (6) | 18 (1) | 1258 |
| 63 | 730 (70) | 126 (12) | 78 (7) | 91 (9) | 20 (2) | 1045 |
| 67 | 587 (62) | 140 (15) | 69 (7) | 126 (13) | 30 (3) | 952 |
| Total | 3773 (75) | 580 (11) | 272 (5) | 348 (8) | 80 (1) | 5053 |

$P < 0.001$ from a Pearson $\chi^2$ test with 12° of freedom.

consent, a venous blood sample of 15 ml was drawn in a heparinized Vacutainer tube. After separations, serum was frozen at – 20°C and sent to the Clinical Laboratory of the Department of Gynaecology and Obstetrics, Helsinki University Hospital. PSA determinations were performed using the Tandem-E assay (Hybritech, San Diego, CA, USA).

In order to detect cancers among men with PSA concentrations below 4.0 ng ml$^{-1}$ and to improve sensitivity, men with serum PSA concentration 3.0–3.9 ng ml$^{-1}$ were offered a supplementary digital rectal examination (DRE) by a urologist. Initially, 119 men with PSA 2.0–2.9 were also offered a DRE examination, but this was soon discontinued to avoid unnecessary examinations. If the DRE finding was suspicious for cancer, the man was referred to a urology clinic for diagnostic examinations.

Men with serum PSA concentration of 4.0 ng ml$^{-1}$ or higher, as well as those with a suspicious DRE finding, were referred to one of the four participating hospitals for diagnostic examinations consisting of DRE, transrectal ultrasound (TRUS) and transrectal Tru-cut biopsy of the prostate. Random sextant biopsies were performed on all men, and additional directed biopsies were done if there were suspicious lesions in DRE, TRUS or both.

If a prostate cancer was diagnosed, staging was conducted using primarily TRUS and bone scan, but also bone X-rays, computed tomography and magnetic resonance imaging when necessary. Similar treatment protocols were used for screen-detected cancers as for other patients given age, grade and stage. The treatment options for organ-confined disease include radical prostatectomy, radical radiotherapy or watchful waiting. Advanced disease is treated with endocrine therapy (anti-androgen, luteinizing hormone releasing hormone agonist, oestrogen or orchidectomy) or watchful waiting.

The study was approved by the ethical committees in each participating hospital.

**Table 3** Distribution of screen-detected prostate cancers by grade, age and serum PSA concentration in the Finnish prostate cancer screening trial

| | Grade (WHO) | | | | | | | |
|---|---|---|---|---|---|---|---|---|
| | I | | II | | III | | Total | |
| | *N* | (%) | *N* | (%) | *N* | (%) | *N* | |
| Age (years) | | | | | | | | |
| 55 | 10 | (50) | 10 | (50) | – | (–) | 20 | |
| 59 | 10 | (29) | 21 | (62) | 3 | (9) | 34 | |
| 63 | 12 | (44) | 15 | (56) | – | (–) | 27 | |
| 67 | 17 | (46) | 13 | (35) | 7 | (19) | 37 | $P$=0.04[1] |
| PSA (ng/ml) | | | | | | | | |
| 2.0–2.9 | 3 | (100) | – | (–) | – | (–) | 3 | |
| 3.0–3.9 | 4 | (44) | 5 | (56) | – | (–) | 9 | |
| 4.0–9.9 | 27 | (43) | 32 | (51) | 4 | (6) | 63 | |
| ≥10 | 15 | (35) | 22 | (51) | 6 | (14) | 43 | $P$=0.27[1] |
| Total | 49 | (42) | 59 | (50) | 10 | (8) | 118 | |

[1]Statistical significance from an exact Pearson $\chi$2 test with 6° of freedom.

### RESULTS

The number of men randomized to the screening arm of the trial was 7337. Of them, 5053 (69%) participated (Figure 1). The participation rate was slightly higher in the Tampere area than in Helsinki, and there were little differences by age (Table 1).

Of the participants, 3773 (75%) had serum PSA concentrations below 2.0 ng ml$^{-1}$, 852 (17%) between 2.0 and 3.9 ng ml$^{-1}$, and 428 (8.5%) 4.0 ng ml$^{-1}$ or higher (Figure 1). The PSA concentration increased with age (Table 2).

In the initial phase of the study, 119 men with PSA between 2.0 and 2.9 ng ml$^{-1}$ were offered a DRE. Of them, 110 (92%) underwent DRE and eight findings (7%) raised suspicion of cancer. Three cancers were diagnosed, yielding a positive predictive value of 38% and detection rate of 2.7% for DRE among men with PSA 2.0–2.9 ng ml$^{-1}$ (Table 3).

The 272 subjects with PSA concentrations of 3.0–3.9 ng ml$^{-1}$ were offered a DRE and 257 (94%) accepted. Among them, 39 men (15%) had a DRE finding suspicious for cancer and were referred to diagnostic examinations. Nine cancers were detected corresponding to a positive predictive value of 23% and detection rate of 3.3% for DRE among men with PSA 3.0–3.9 ng ml$^{-1}$ (Table 3).

All 428 men with a serum PSA concentration of 4.0 ng ml$^{-1}$ or higher were referred to diagnostic examinations including DRE, TRUS and prostate biopsy. Of them, 27 did not comply, because they were already being treated by another urologist ($n$ = 10), had moved from the study area ($n$ = 3), had died ($n$ = 2) or declined further examinations ($n$ = 12). Of the 401 men evaluated, 106 were diagnosed with prostate cancer (Table 3). Hence, the positive predictive value was 26% for PSA of 4.0 ng ml$^{-1}$ or higher, and 54% for PSA of 10 ng ml$^{-1}$ or higher. The positive predictive value

© Cancer Research Campaign 1999 

**Table 4** Distribution of screen-detected cancers by clinical stage, age and serum prostate-specific antigen (PSA) concentration, Finnish prostate cancer screening trial

| | Clinical stage | | | | |
|---|---|---|---|---|---|
| | **T1N×M0** *N* (%) | **T2N×M0** *N* (%) | **T3–4N×M0/ T1–4N×M1** *N* (%) | **Total** *N* | |
| Age (years) | | | | | |
| 55 | 7 (35) | 11 (55) | 2 (10) | 20 | |
| 59 | 13 (38) | 15 (44) | 6 (18) | 34 | |
| 63 | 11 (41) | 14 (52) | 2 (7) | 27 | |
| 67 | 13 (35) | 16 (43) | 8 (22) | 37 | *P*=0.78[1] |
| PSA (ng/ml) | | | | | |
| 2.0–2.9 | – (–) | 3 (100) | – (–) | 3 | |
| 3.0–3.9 | – (–) | 9 (100) | – (–) | 9 | |
| 4.0–9.9 | 32 (51) | 26 (41) | 5 (8) | 63 | |
| ≥10.0 | 12 (28) | 18 (42) | 13 (30) | 43 | *P*>0.001[1] |
| Total | 44 (37) | 56 (48) | 18 (15) | 118 | |

[1]Statistical significance from an exact Pearson $\chi 2$ test with 6° of freedom.

of serum PSA concentration of 4.0 ng ml$^{-1}$ or higher decreased from 31% among men less than 60 years of age to 22% among men older than 60 years (data not shown).

The detection rate attributable to PSA concentration of 4.0 ng ml$^{-1}$ or higher alone was 2.1% among study participants. When DRE examinations among men with PSA below 4.0 ng ml$^{-1}$ were also taken into account, the overall detection rate of the screening programme was 2.3% among participants or 1.6% among all subjects in the screening arm.

Approximately nine out of ten screen-detected tumours were well or moderately differentiated (Table 3) and organ-confined (Table 4). Five subjects had metastatic disease. The grade tended to be slightly lower among younger age groups. There were no major differences in the stage distribution by age. As expected, the proportion of organ-confined tumours was higher among men with PSA below 10 ng ml$^{-1}$ than among men with higher PSA values.

Only preliminary information on tumour volume, pathological staging and treatment outcomes was available at this time and these preliminary end points will therefore be reported separately later on.

### DISCUSSION

Our results indicate that good participation rate can be obtained in a population-based screening study of prostate cancer screening. The positive predictive value using PSA cut-off level of 4.0 ng ml$^{-1}$ is acceptable. Furthermore, the vast majority of screening-detected cancers are well or moderately differentiated and organ confined.

The Finnish prostate cancer screening trial is population based, i.e. we were able to define the study base and identify all subjects in it. Compared with volunteer-based studies, the population-based approach has the advantage of permitting estimation of effects in the general population, i.e. screening implemented as health care policy. This requires that the screened subjects are representative of the population. We had a high participation rate (69%), which is essential to fulfil this requirement. The control group was not subject to any intervention. Thus, an informed consent was not required. However, information on prostate cancer incidence and mortality in the control group at the aggregate level can be obtained through record-linkage. Prostate cancer among men from both the screening and the control arm are treated almost exclusively at the participating clinics. Hence, similar treatment for both groups can be assured and bias from different policies or outcomes of treatment avoided.

The results presented here are based on intermediate end points including participation rate, positive predictive value of the test, detection rate, and characteristics of screen-detected cancers. The primary end points of the study are mortality from prostate cancer and quality of life. A prerequisite for mortality reduction is that a larger proportion of screen-detected cancers are curable than of cancers detected otherwise. For prostate cancer, this means that cancers should be detected and treated at organ-confined stage. However, improved stage distribution could also result from increased detection of indolent cancers. Therefore, improvement of stage distribution compared with otherwise-detected cancers is a necessary, but not sufficient proof of effectiveness of a screening programme. In general, a screening programme cannot be evaluated on the basis of intermediate end points alone. Prostate cancer screening cannot be recommended for the general population until there is evidence of mortality reduction, and lack of adverse quality of life effects as well as reasonable cost-effectiveness have been demonstrated.

The positive predictive value for PSA with cut-off level of 4.0 ng ml$^{-1}$ was 27%, which is comparable with mammography screening (Hakama et al, 1991; United Kingdom Trial of Early Detection of Breast Cancer Group, 1992; Kerlikowske et al, 1993). It is possible that the positive predictive value could be further improved by determining the free PSA concentration in addition to the total PSA (Lilja et al, 1991; Stenman et al, 1991, 1994). The detection rate for digital rectal examination among men with PSA below 4.0 ng ml$^{-1}$ was an order of magnitude lower than among men with PSA levels above 4 ng ml$^{-1}$, which is not very encouraging. In fact, the detection rate for men with PSA in the range 2.0–3.9 ng ml$^{-1}$ (3.1%) was close to that of the whole study population regardless of PSA concentration (2.3%), which suggests that men with this PSA level do not have a materially increased risk of prostate cancer compared with the general population. The ERSPC trial protocol is based on total PSA concentration, but it is possible

 *© Cancer Research Campaign 1999*

that incorporation of free to total PSA could decrease the number of false-positive test results and improve the specificity of the screening protocol. This issue is being investigated.

The overall detection rate of prostate cancer during the first year of the Finnish trial (approximately 2%) is practically identical with previous results from studies using a similar screening algorithm (Catalona et al, 1991). Studies with other screening modalities combined with PSA have had higher detection rates (Mettlin et al, 1996; Schröder et al, 1996). Generally, lower detection rates have been reported for subsequent screens following the first round (Labrie et al, 1996; Smith et al, 1996).

The detection rate is higher than in mammography screening (Day et al, 1988; Hakama et al, 1991; United Kingdom Trial of Early Detection of Breast Cancer Group, 1992). This can be expected given the relatively slow mean growth rate of prostate cancer (Schmid et al, 1993; Stenman, 1997) compared with breast cancer (Peer et al, 1993).

In our study, approximately nine out of ten screen-detected cancers were well or moderately differentiated and organ confined. This is comparable with other studies (Labrie et al, 1996; Mettlin et al, 1996; Smith et al, 1996; Schröder et al, 1996). Screen-detected cancers were detected substantially more frequently at a curable stage (85% vs 34% clinically organ confined) than other prostate cancers (Auvinen et al, 1998). Typically, a larger proportion of well-differentiated, slowly growing tumours are detected in the first (prevalence) screening round than subsequent (incidence) screening rounds, because of length bias (Hakama, 1991). Hence, the comparison of tumour characteristics should ideally be conducted between cancers diagnosed in the screening arm during the prevalence screen, those diagnosed at subsequent screening rounds and those in the control arm. This is, however, not possible until completion of the second screening round. Empirical results pertaining to possible overdiagnosis due to screening will have to wait until sufficient data from randomized trials are available.

A critical issue in prostate cancer is which cancers should be treated. Latent prostate cancer is a common incidental finding at autopsy and prostate surgery (Breslow et al, 1977; Potosky et al, 1990). Small, well-differentiated cancers detected in asymptomatic men are regarded as clinically insignificant (Dugan et al, 1996). A screening programme, however, is intended to detect cancers at a pre-clinical stage, when they are small and probably well-differentiated. It is difficult to project tumour size and grade if the cancers were detected later, possibly at a symptomatic stage. Therefore, it is unclear whether similar criteria of clinical significance can be applied to screening-detected cancers. Longitudinal population studies of serum PSA suggest that most prostate cancers grow at a slow, but steady rate (Schmid et al, 1993; Stenman, 1997). A tumour with a doubling time of 2 years will grow from 1 g to about 32 g in 10 years and to 1 kg, which is likely to be lethal, in 20 years.

The Finnish trial is designed as a low-intensity intervention: the cut-off level for PSA is higher and screening interval longer than in most other studies. This approach was chosen to minimize both costs and potential adverse effects. Low overall cost could also mean better cost-effectiveness given that the mortality reduction and quality of life effects are comparable with more intensive screening regimens.

The Finnish trial is a part of the ERSPC. The target sample size of the European study is more than 180 000 subjects. This would be sufficient to detect an average mortality reduction of 17% at 10 years of follow-up. A joint analysis is also planned with the European and North American studies, which provides even better power (Auvinen et al, 1996). Non-randomized studies will not provide information on mortality reduction, due to lack of a valid control group. Quality of life effects are an equally important issue and will be assessed as part of the Finnish trial.

In summary, the first results of the Finnish prostate cancer screening trial suggest that a good participation rate can be achieved in a population-based screening trial, the positive predictive value of a PSA concentration above 4 ng $ml^{-1}$ is comparable with a positive mammography result, and the cancers detected have characteristics associated with more favourable prognosis compared with tumours detected otherwise.

### ACKNOWLEDGEMENTS

We thank the Academy of Finland, Cancer Society of Finland, Helsingin Sanomat Centenarian Foundation, Tampere University Hospital Research Fund, Finnish Cultural Fund, Hybritech Corporation and Europe Against Cancer programme for financial support. We are grateful to Pirkanmaa Cancer Society and Aleksi Medical Centre for drawing blood samples for the study, Esko T Voutilainen BSc for programming and Ms Minna Heikkilä for data entry.

### REFERENCES

Auvinen A, Rietbergen JBW, Denis LJ, Schröder FH and Prorok PC (1996) A prospective evaluation plan for randomised trials of prostate cancer screening. *J Med Screening* **3**: 97–104

Auvinen A, Hakama M, Vornanen T, Leppilahti M, Ala-Opas M, Salminen P and Tammela T (1998) A randomised trial of choice of treatment in prostate cancer (submitted for publication)

Breslow NE, Chan CW, Dhom G, Drury RAB, Franks LM, Gellei B, Lee YS, Lundberg S, Sparke B, Sternby NH and Tulinius H (1977) Latent carcinoma of prostate at autopsy in seven areas. *Int J Cancer* **20**: 680–688

Catalona WJ, Smith DS, Ratliff TL, Dodds KM, Coplen DA, Yuan JJ, Petros JA and Andriole GL (1991) Measurement of prostate-specific antigen in serum as screening test for prostate cancer. *N Engl J Med* **324**: 1156–1161

Day NE, Walter SD, Tabár L, Fagerberg CJG and Collette HJA (1988) The sensitivity and lead time of breast cancer screening: a comparison of the results of different studies. In *Screening for Breast Cancer*. Day NE and Miller AB (eds). International Union Against Cancer and Hans Huber Publishers; Stuttgart

Denis L, Murphy GP and Schröder FH (1995) Report of the consensus workshop on screening and global strategy for prostate cancer. *Cancer* **75**: 1187–1207

Dugan JA, Bostwick DG, Myers RP, Qin J, Bergstrahl EJ and Oesterling JE (1996) The definition and preoperative prediction of clinically insignificant prostate cancer. *J Am Med Assoc* **275**: 288–294

Hakama M (1991) Screening. In *Oxford Textbook of Public Health*, pp. 91–106. Oxford University Press: Oxford

Hakama M, Elovainio L and Louhivuori K (1991) Breast cancer screening programme in Finland. *Br J Cancer* **64**: 962–964

Kerlikowske K, Grady D, Barclay J, Sickles EA, Eaton A and Ernster V (1993) Positive predictive value of screening mammography by age and family history. *J Am Med Assoc* **270**: 2444–2450

Labrie F, Candas B, Cusan L, Gomez JL, Diamond PS, Suburu R and Lemay M (1996) Diagnosis of noncurable prostate cancer can be practically eliminated by prostate specific antigen. *Urology* **47**: 212–217

Lilja H, Christenssen A, Dahlen U, Matikainen MT, Nilsson O, Pettersson K and Lövgren T (1991) Prostate-specific antigen in serum occurs predominantly in complex with alpha-1-antichymotrypsin. *Clin Chem* **37**: 1618–1625

Mettlin C, Murphy GP, Babaian RJ, Chesley A, Kane RA, Littrup PJ, Mostofi FK, Ray PS, Shanberg AM and Toi A (1996) The results of a five-year early prostate cancer detection intervention. *Cancer* **77**: 150–159

Parkin DM, Whelan SL, Ferlay J, Raymond L and Young JL Jr (eds) (1997) *Cancer Incidence in Five Continents, Vol. VII*. International Agency for Research on Cancer: Lyon

Peer PGM, Van Dijck JAAM, Hendriks JHCL, Holland R and Verbeek ALM (1993) Age-dependent growth rate of primary breast cancer. *Cancer* **71**: 3547–3551

© Cancer Research Campaign 1999 

Potosky AL, Kessler L, Gridley G, Brown CC and Horn JW (1990) Rise in prostatic cancer incidence associated with increased use of transurethral resection. *J Natl Cancer Inst* **82**: 1624–1628

Schmid H-P, McNeal JE, Stamey TA (1993) Observations on the doubling time of prostate cancer. *Cancer* **71**: 2031–2040

Schröder FH and Boyle P (1993) Screening for prostate cancer – necessity or nonsense? *Eur J Cancer* **29A**: 656–661

Schröder FH, Damhuis RAM, Kirkels WJ, De Koning HJ, Kranse R, Nisj HGT, Blijenberg BG (1996) European randomized study of screening for prostate cancer – the Rotterdam pilot studies. *Int J Cancer* **65**: 145–151

Smith DS, Catalona WJ and Herschman JD (1996) Longitudinal screening for prostate cancer with prostate specific antigen. *J Am Med Assoc* **276**: 1309–1315

Stenman U-H, Leinonen J, Alfthan H, Rannikko S, Tuhkanen K and Alfthan O (1991) A complex between prostate-specific antigen and alpha-1-antichymotrypsin is the major form of prostate-specific antigen in serum of patients with prostate cancer: assay of the complex increases clinical sensitivity for cancer. *Cancer Res* **51**: 222–226

Stenman U-H, Hakama M, Knekt P, Aromaa A, Teppo L and Leinonen J (1994) Serum concentrations of prostate specific antigen and its complex with α1-antichymotrypsin before diagnosis of prostate cancer. *Lancet* **344**: 1594–1598

Stenman U-H (1997) Prostate-specific antigen, clinical use and staging: an overview. *Br J Urol* **79** (Suppl 1): 53–60

Teppo L, Pukkala E and Lehtonen M (1994) Data quality and quality control of a population-based cancer registry. *Acta Oncol* **33**: 365–369

United Kingdom Trial of Early Detection of Breast Cancer Group (1992) Specificity of screening in United Kingdom trial of early detection of breast cancer. *Br Med J* **304**: 346–349

*© Cancer Research Campaign 1999*
